# Analyzing Gait in the Real World Using Wearable Movement Sensors and Frequently Repeated Movement Paths

**DOI:** 10.3390/s19081925

**Published:** 2019-04-24

**Authors:** Weixin Wang, Peter Gabriel Adamczyk

**Affiliations:** Department of Mechanical Engineering, University of Wisconsin–Madison, Madison, WI 53706, USA; wwang442@wisc.edu

**Keywords:** wearable sensor, gait analysis, gait variability, location tracking, pedestrian dead-reckoning

## Abstract

Assessing interventions for mobility disorders using real-life movement remains an unsolved problem. We propose a new method combining the strengths of traditional laboratory studies where environment is strictly controlled, and field-based studies where subjects behave naturally. We use a foot-mounted inertial sensor, a GPS receiver and a barometric altitude sensor to reconstruct a subject’s path and detailed foot movement, both indoors and outdoors, during days-long measurement using strapdown navigation and sensor fusion algorithms. We cluster repeated movement paths based on location, and propose that on these paths, most environmental and behavioral factors (e.g., terrain and motivation) are as repeatable as in a laboratory. During each bout of movement along a frequently repeated path, any synchronized measurement can be isolated for study, enabling focused statistical comparison of different interventions. We conducted a 10-day test on one subject wearing athletic shoes and sandals each for five days. The algorithm detected four frequently-repeated straight walking paths with at least 300 total steps and repetitions on at least three days for each condition. Results on these frequently-repeated paths indicated significantly lower foot clearance and shorter stride length and a trend toward decreased stride width when wearing athletic shoes vs. sandals. Comparisons based on all straight walking were similar, showing greater statistical power, but higher variability in the data. The proposed method offers a new way to evaluate how mobility interventions affect everyday movement behavior.

## 1. Introduction

Treatment for musculoskeletal mobility disorders includes many products and rehabilitation strategies, but there is little sound assessment about how these affect individuals in their daily lives. Current research assessing outcomes and developing standards of care is based on either (i) focused, laboratory-based studies, or (ii) studies based on wearable sensors such as pedometers, accelerometers, and heart rate monitors in everyday life. Both approaches have drawbacks that make them inadequate for fully assessing the effects of interventions on mobility. Laboratory tests enable well-controlled comparisons of detailed data, but also impose unrealistic influences on subjects, such as social pressure to perform well, heightened attention, instructions for the walking task, etc. [1]. Furthermore, these tests require extensive infrastructure and are only available in limited locations to a small number of individuals [2]. On the other hand, long-term monitoring through field-based wearable sensors provides a minimally-altered window into everyday movement [2], but these data can be difficult to distill into generalizable knowledge because many influencing factors are left uncontrolled, such as terrain, weather, setting, and purpose of locomotion. Neither approach enables a thorough assessment of how interventions influence individuals’ movement. 

In this paper, we propose an assessment method that combines the strengths of a controlled laboratory study and those of a more realistic wearable measurement in daily life. The key idea is to focus the wearable assessment on the most informative and repeatable everyday activities, and make a detailed comparison of movement during these specific activities. Our approach recognizes that a days-long record of movement may include a variety of behaviors including walking, standing, sitting, running, and more, which may be performed in a variety of settings including homes, offices, parks, sidewalks, stores, and others [3]. This variety calls into question the validity of comparisons that include all movements without checking for comparable conditions. Our core hypothesis is that this variety leads to high variability in movement metrics, which can be reduced by focusing on frequently-repeated movements. Our goal is to provide a method to identify these repeated movements and thereby eliminate irrelevant or confounding data, leaving a subset of highly repeatable movements that can be analyzed with laboratory-like confidence. The resulting analysis is intended for use in comparing mobility interventions such as different prostheses, orthoses, or rehabilitation programs to establish their relative effectiveness and clinical value. 

### 1.1. Studying Mobility with Wearable Sensors 

Most approaches to studying movement with wearable movement sensors fall into two categories: (i) Comprehensive movement studies using sensors mounted on multiple individual segments of the body, and (ii) studies of bulk motion using single sensors, usually mounted on the waist. Multi-segment sensor networks estimate detailed motion of the skeleton using inertial sensors on different segments and an underlying skeleton model (e.g., XSens MVN [4] or LegSys [5]). Since movement sensors do not measure ground forces, they have sometimes been combined with force sensors in the shoes [6,7,8]. These techniques enable lab-like analysis using wearable sensors, but are inappropriate for long-term monitoring due to the need for daily mounting and charging of many sensors—from 5 to 17 or even more. Alternatively, single-sensor approaches typically focus on estimating macro-scale behavior such as number of walking steps or overall activity level; these measures can be informative in some circumstances [3,9,10,11,12], but can also be insensitive to changes in movement capacity or satisfaction [13,14]. Some single-sensor studies target finer gait characteristics like step length, turning frequency, or disease-specific outcomes [12,13,15,16], but these approaches still lack detail about foot and leg movement that can be important for understanding underlying biomechanical function. Efforts to focus on a middle-ground of achieving low burden to the user with relatively fine measured detail have focused on reconstructing gait kinematics from shoe-mounted inertial sensors [1,17,18,19,20,21,22]. This motion reconstruction provides a high-fidelity, model-free estimate of foot motion that can form the basis for analyses of gait stability and control and even losses-of-balance [1,23,24]. 

All these approaches are subject to significant scientific limitations when used to compare mobility conditions or experimental interventions using everyday data. Daily life contains a wide variety of activities [3], which may not all demonstrate the same movement characteristics. For example, walking to the morning bus is different from walking in a grocery store—different in purpose, urgency, terrain, surroundings, interactions, carried load, and more. In continuous monitoring with wearable sensors, such different contexts add variability to the recorded data. Furthermore, unique activities like participation in tours, hikes, or festivals could also be recorded, and existing analytical approaches would have no means of rejecting them. In the worst case, these real-world data sets could be so inclusive that their variability obscures important differences among mobility conditions. Even when they do differentiate, there is no way to be sure that observed differences are attributable to the conditions, rather than the uncontrolled circumstances of the data collection. 

### 1.2. Laboratory-Like Mobility Analysis on Frequently-Repeated Paths 

Fortunately, within the great variability of everyday movement, certain components of the daily routine are repeated as regularly as controlled laboratory conditions. For example, an individual may walk from the same home to the same bus stop, or from the same parking lot to the same building entry, or from the same workstation to the same break room, every day or even several times per day. Moreover, the motivation, terrain, and location are similar for these movements nearly every time they occur. This similarity suggests that in these frequently-repeated circumstances, most of the behavioral factors are as repeatable as in a controlled laboratory study. In this paper we propose a method to identify and analyze these frequently-repeated natural movements to assess the effects of different experimental interventions on gait. The paper describes the method and applies it to a simple comparison of two types of footwear, with the intention of future application to clinical mobility interventions.

In the proposed method we use location data to isolate repeated paths from the person’s movement trajectory during days-long wearable recordings. The trajectory is reconstructed from global positioning system (GPS) location data, foot motion data from a shoe-mounted inertial movement sensor, and altitude data from a barometric pressure sensor. Data from all these sensors are combined through strapdown inertial navigation and sensor fusion algorithms. During each bout of movement along a frequently-repeated path, we isolate spatiotemporal parameters of walking [1,17,18,19,25] and any other synchronous measurements. Finally, we compare these measurements across different conditions of interest, such as before and after some form of intervention, or across multiple interventions such as different assistive devices. This approach enables the study of how these interventions affect everyday movement, without the challenges of a laboratory environment.

## 2. Materials and Methods

### 2.1. Overview

The trajectory reconstruction method is based on a standard inertial navigation algorithm termed pedestrian dead-reckoning (PDR), which uses a shoe-mounted inertial measurement unit (IMU, including a 3-axis accelerometer, a 3-axis angular rate gyroscope, and optionally a 3-axis magnetometer). The algorithm integrates angular velocity and linear acceleration into position, and corrects drift errors using zero-velocity update (ZUPT) [26], zero-angular-rate update (ZARU) [27], GPS location, and barometric altitude (BA) through a 15-state Kalman smoother [26,28] (details in Appendix B). ZUPT is used to correct velocity and orientation drift by assuming that foot velocity is zero during each stance phase of walking, running, or standing. ZARU uses angular velocity to estimate gyroscope bias during long stationary periods, with the goal of reducing heading drift that cannot be corrected by ZUPT. GPS and BA are used to correct latitude/longitude and altitude errors. The Kalman smoother is used to fuse all available sensor readings and assumptions into a maximum likelihood estimate of the user’s location. The different corrections are fed into the Kalman smoother based on specific movement conditions, listed in Table 1 and described below.

### 2.2. Using Outdoor GPS Data for Position Corrections

GPS is a good reference for absolute position correction of inertial sensors during outdoor periods in the horizontal plane. In the vertical direction, GPS data is usually less accurate and therefore not usable. On the other hand, because barometric altitude is sensitive to weather conditions and can only be used for relative altitude change, an absolute reference in the vertical direction is still needed. We use altitude data over a grid with 10-m (1/3 arc second) resolution from the USA Geological Survey’s National Elevation Dataset [29] (a component of The National Map [30]) as the absolute altitude reference. This database covers the United States; other similar databases may be used in other areas. For each GPS location fix, the altitude is interpolated from the grid based on the latitude and longitude. 

**Figure 1:**
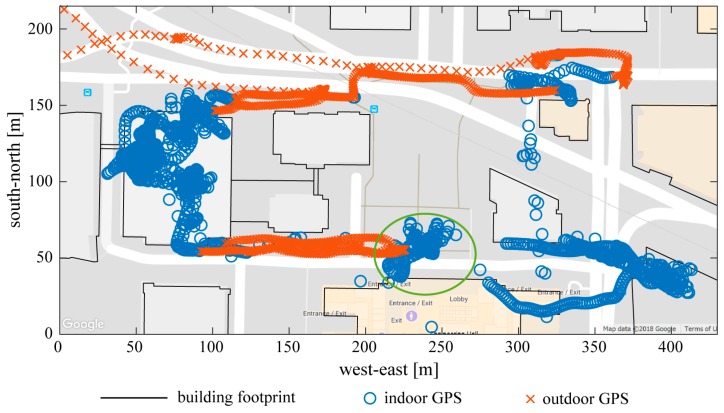
Indoor vs. Outdoor GPS recognition. Indoor GPS locations are indicated by blue circles; outdoor GPS locations are indicated by red crosses. Black lines are building footprints downloaded from OpenStreetMap. Indoor GPS locations are not necessarily within a building’s footprint, which makes the recognition very challenging. For example, the GPS fixes in the green ellipse are in fact inside the building on their south; using these erroneous data for position correction would ruin the indoor trajectory reconstruction.

In addition, GPS accuracy degrades greatly inside of buildings and therefore cannot be used for indoor position correction. The challenge of using GPS data is that a GPS receiver can often receive a location fix indoors, but we have very limited knowledge of its accuracy; thus, it is very difficult to separate it from usable outdoor location fixes. Moreover, even if a receiver sits still inside of a building, its location fixes can wander, including to locations that appear to be outdoors on the map. In our method, using an erroneous GPS location for indoor position correction can be destructive, while recognizing outdoor locations as indoor without position correction merely degrades the trajectory accuracy for a short time. Therefore our algorithm is designed to avoid the first circumstance as much as possible to maintain an overall acceptable accuracy. Generally, outdoor GPS is characterized by: (i) Low horizontal dilution of precision (HDOP, a measure of GPS sensitivity to signal timing error); (ii) small time gaps between consecutive location fixes (e.g., a few seconds); (iii) small spatial separation between consecutive location fixes; and (iv) location fixes that do not gather near a single building’s footprint. We used these characteristics to build a rule-based classification for recognizing indoor vs. outdoor GPS; details are presented in Appendix A and an example is shown in Figure 1. Building footprints were obtained from the open source database OpenStreetMap [31], which is available in many geographical areas. 

### 2.3. Special Movement Conditions for Different Corrections

During outdoor periods, we also detect periods of vehicle use, which are defined by (i) high GPS speed and (ii) absence of large and long-lasting IMU movements. In a vehicle, ZUPT is not applied since the velocity of the IMU is not zero, but GPS updates are sufficient to correct position drift. During indoor periods, special conditions such as stationary periods and elevators provide additional corrections and challenges. Stationary conditions are defined by long-lasting low angular velocity, such as while sitting or standing at a workstation. We use these stationary conditions to adjust the gyroscope bias using measured angular velocity. Elevator use is detected by a rapid change in barometric altitude without large movements indicated by the IMU. In this case, the assumption of zero foot velocity is violated in the vertical direction, so we use relative BA for vertical position correction. These special movement conditions can be detected by proper thresholding on the respective signals mentioned above.

### 2.4. Indoor Heading Angle Correction

Outdoors, heading angle can be corrected by a series of GPS measurements, but because GPS is not reliable indoors it cannot correct heading inside buildings. Indoors, ZUPT is the primary correction, but because heading error is only weakly correlated to velocity in stance phase [26,27], ZUPT is not adequate to stabilize heading over long durations. Without adequate correction, sensor bias and numerical integration errors lead to the build-up of unbounded errors in heading and consequently in the estimated location. The magnetometer available in some IMUs does give a heading measurement, but magnetic field fluctuations due to metallic objects (beams, pipes, wires, etc.) make this measurement practically unusable indoors. Therefore, another means is required to correct sensor heading and estimate position over the long indoor periods in a typical day. 

**Figure 2:**
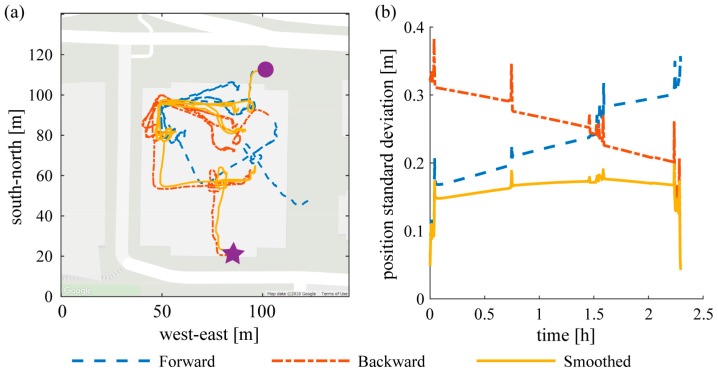
Comparison of (**a**) forward (blue dashed line), backward (red dash-dot), and smoothed (yellow solid) position and (**b**) their standard deviations during a 2+ hour indoor period. The subject entered the building from the north (round dot on the left graph), stayed more than 2 h in the building and left from the south (star on the left graph). It can be seen that forward filter’s position is accurate at first but gradually drifts counter-clockwise; the backward filter’s position is accurate at the end but drifts clockwise in reverse time. The smoothed position follows the forward position at first and gradually shifts to the backward position, maintaining a smaller standard deviation throughout. Note that the standard deviations on the right graph are estimates from the Kalman filter and smoother, where only their relative magnitude is important.

We address this problem by using a Kalman smoother (Appendix B) to propagate GPS data both forward and backward in time, thereby reducing the error growth time to half of each continuous period spent indoors. The Kalman smoother uses the same Kalman filter twice: once forward and once backward in time. A final position estimate is produced by averaging the two one-way estimates, each weighted by its uncertainty [28], which grows with the amount of time since the last GPS update. The forward filter is more accurate at first because of short integration time after the loss of GPS. Thus, the estimated position variance is lower, meaning forward position contributes more to the smoothed position. The forward Kalman filter becomes less accurate as the integration time grows, and its estimated position variance grows with it; simultaneously the backward Kalman filter time shrinks (time until the next future GPS update) and its estimated position variance also reduces. As a result, the forward position estimate contributes less to the smoothed position as GPS-denied periods progress, and the backward estimate contributes more (Figure 2). The smoothed position is statistically the best estimate in terms of the least mean squared errors.

### 2.5. Finding Repeated Paths 

**Figure 3:**
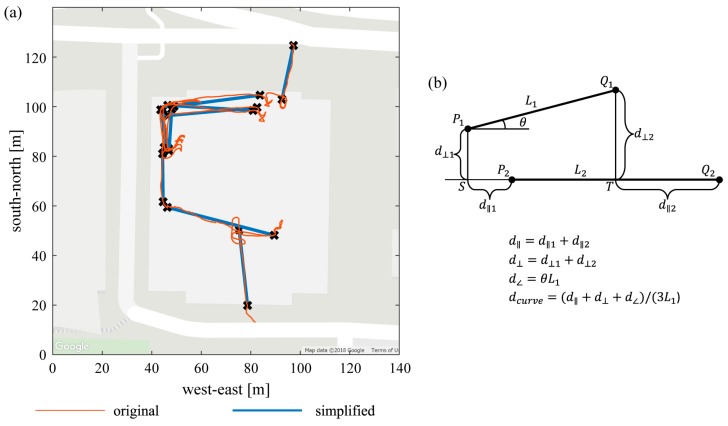
Curve simplification and distance. (**a**) Simplification result of a sample path: the original trajectory before discretization is indicated by the thin red curve, the straight segments separated from the original curve are indicated by bold blue lines and black markers. Typical straight walking trajectories in hallways are singled out after curve simplifications. (**b**) Definition of “curve distance”, for assessing spatial separation of two paths, lengths L1 (shorter) and L2 (longer). Curve distance is a composite of: Parallel distance (distance between two segments’ starting points along the longer path); perpendicular distance (distance of the start and end of L1 from the line of L2); and angular distance (angle between path directions, including progress direction, times L1).

To find repeated movements of the person, we first discretize the reconstructed trajectory by stance phases during walking and by intermittent GPS waypoints when the subject is in a vehicle. We then simplify the discretized trajectory using the Ramer-Douglas-Peucker algorithm [32] in which the distance parameter is set to 1 m. This algorithm combines sequential location readings to reduce the point density of a trajectory. Then we use a modified Visvalingam’s algorithm [33] to further simplify the trajectories and identify straight segments. The modified Visvalingam’s algorithm recursively deletes any point at which the angle formed by itself and its two neighbors is above a specified “straightness” threshold (here set to 2.85 radians). Segments longer than a specified length threshold (here 15 m) are picked out as straight walking trajectories. An example of an original trajectory and its straight-segment approximation is shown in Figure 3a.

Within the resulting set of straight trajectory segments, we identify groups of segments that occur in the same location and walking direction. We define a curve distance incorporating parallel, perpendicular and angular differences [34] (see Figure 3b) to evaluate the spatial separation between each pair of segments. Segments in the same location are grouped into a cluster such that for each segment, there is at least one other segment in the same cluster for which the distance between them is within a threshold (here set to 0.2, dimensionless). We retain clusters only if they contain enough strides to make a robust statistical comparison across conditions (here 300 strides per condition with occurrences on at least three days per condition). Future work could elaborate this and other curve simplification and matching techniques to identify other trajectories of interest, such as stairs, turning, specific locations, etc. 

### 2.6. Statistical Comparison of Conditions along Repeated Paths 

For statistical analysis, gait parameters and other measures of interest are pooled by condition for each cluster of path segments. Here, we reconstruct foot motion anew for each segment in the remaining clusters, using only acceleration and angular velocity data from the IMU to avoid any discontinuity or distortion due to GPS or BA corrections. We compute stride length, stride speed, and stride width from the reconstructed trajectories using the method described in Ref. [1]. We further compute stride clearance as the minimum height during swing phase above a line connecting successive footfalls. We pool these measures for each experimental condition and compare across conditions. Any other synchronized measurement (e.g., heart rate, temperature, perspiration, muscle activity, tremor, etc.) could be similarly segmented and pooled based on the repeated paths. 

As many gait parameters and mobility metrics change systematically with the behavioral factor of walking speed, we first check each parameter for such dependence by computing its linear regression against walking speed. If no significant relationship is found (*p*-value of slope > 0.05), we apply a two-sample t-test or Analysis of Variance (ANOVA) to compare the mean values of the parameter across different conditions. If the slope of the regression is significant, we apply Analysis of Covariance (ANCOVA) to estimate the difference across conditions using population marginal means and their variances at the mean speed. 

### 2.7. Preliminary Testing 

**Figure 4:**
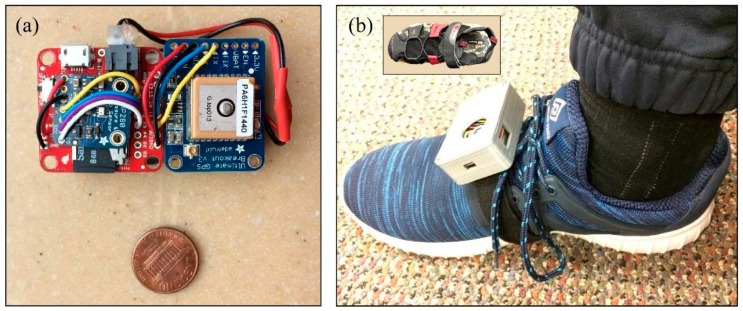
Sensor system used for the pilot study. (**a**) The sensors include a full IMU (9Dof Razor IMU M0, Spark Fun Electronics, including an InvenSense MPU-9250 IMU chip), a barometric pressure sensor (Adafruit Electronics breakout for Bosch BMP280 chip), and a GPS receiver (Adafruit Electronics Ultimate GPS Breakout version 3, using MediaTek Labs MTK3339 chip). GPS data include a time stamp used to synchronize the embedded system with enhanced location data collected on a smart phone (not shown). The sensors are powered by an 850 mAh 1-cell lithium polymer battery that lasts roughly 17 h (behind the sensors in photograph). (**b**) The sensor package (70 × 48 × 26 mm; 69 g including battery) is small and light enough to avoid impeding movement; future versions are expected to be even smaller with custom circuitry. It is mounted on the foot using a strap of hook-and-loop material. The system is recharged each night using a USB cable. For this preliminary study, the sensor was worn with athletic shoes (shown) and with hiking-style sandals (inset).

We conducted a preliminary test to verify the effectiveness of the method described above in comparing spatially repeated straight walking in different conditions during everyday life. One of the authors wore an integrated sensor system (Figure 4) containing an IMU, a barometric pressure sensor and an embedded GPS receiver on his right foot, and also recorded GPS data on his cell phone. Data were recorded for 10 workdays, with footwear as the explanatory variable of interest: five days each with athletic shoes and hiking-style sandals (Figure 4). The study was conducted according to procedures approved by the University of Wisconsin Institutional Review Board. 

## 3. Results

**Figure 5:**
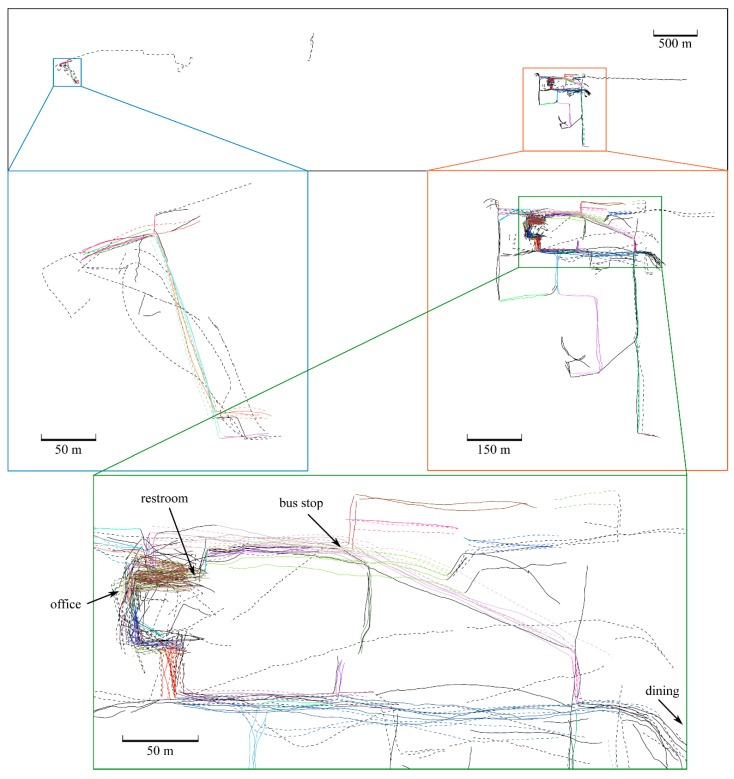
Straight walking trajectories reconstructed during a 10-day walking test, including proper relative geographic location (absolute coordinates removed). The subject wore athletic shoes (solid lines) and sandals (dashed lines) for 5 days each. The figure shows all straight paths longer than 15 m over all 10 days. Matched clusters are shown in different colors, and un-clustered paths are shown in black. The same route traversed in opposite directions counts as two separate paths. Gaps and disconnects in the data arise from several sources, such as: Stationary periods; walking bouts less than 15 m long; corners and other turns; travel by car, bus or bicycle; erroneous path reconstructions; and any other path that did not satisfy the criterion “straight paths longer than 15 m”. Note: this figure is available in vector graphics format in the Supplementary Material for closer study.

The movement reconstruction algorithm rebuilt 48831 total foot displacements over 10 days. Not all foot displacements are walking, as the foot is frequently moved even when sitting or standing in one place. Figure 5 shows the reconstruction of all bouts of straight line walking of 15 m or more, accounting for 27,302 strides (56% of the total foot displacements). Paths that were repeated are shown in matched clusters in color; unique or unmatched paths are shown in black. 

**Figure 6:**
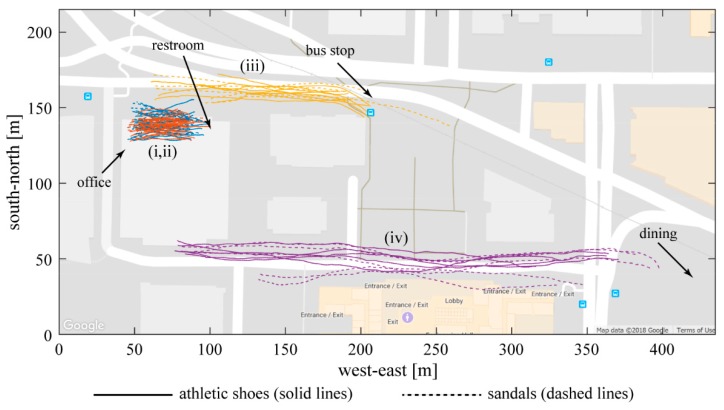
Frequently-repeated straight walking trajectories found from a 10-day walking test. The subject wore athletic shoes and sandals for 5 days each, indicated by solid lines and dashed lines respectively. Matched colors indicate clustered paths with at least 300 strides over at least three days in each condition. These paths all occurred within the green box at the bottom of Figure 5, near the labeled features (colors are changed for greater contrast). Blue and red lines (i,ii) are walking trajectories in the hallway between the subject’s office and the restroom and water fountain (different directions are separated into two different paths). Yellow lines (iii) represent the sidewalk between the building where the subject works and a nearby bus stop. Purple lines (iv) represent a sidewalk between the same building and a dining location.

The algorithm identified four frequently-repeated walking paths with at least 300 strides over at least three days in each condition (Figure 6). These paths are: (i, ii) the hallway between the subject’s office and the restroom and water fountain, in both directions; (iii) the sidewalk connecting the building where the subject works and a nearby bus stop; and (iv) the sidewalk connecting the same building and a dining location. 4811 strides were recorded on these specific paths, accounting for 10% of total foot displacements and 18% of strides in straight-line walking. 

**Figure 7:**
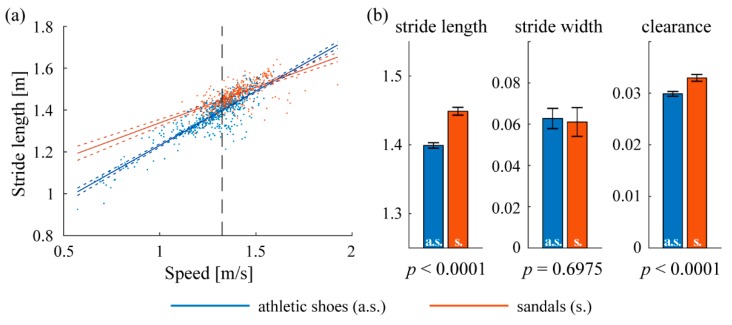
Spatiotemporal gait parameters on path (ii) (Figure 6), the hallway from the subject’s office to the restroom and drinking fountain. Blue and red indicate wearing athletic shoes (a.s.) and sandals (s.), respectively. (**a**) Linear regression of stride length on walking speed. Dotted lines represent 95% confidence intervals and the vertical dashed line represents the mean speed (1.32 m/s) where population marginal means of the two conditions are compared. The slopes of both fitted lines are significantly different from zero (*p* < 0.0001), indicating significant dependence on speed. Thus, ANCOVA is used for the comparison to account for the differences caused by different speeds. (**b**) Population marginal means of stride length, stride width and clearance; error bars indicate 95% confidence intervals.

Stride length wearing athletic shoes was significantly shorter on all repeated paths (mean 1.42 vs. 1.47 m, *p* < 0.0001). Stride width was not different (*p* > 0.05) on three of the four paths, with one exception on the hallway from the restroom to the subject’s office where stride width wearing athletic shoes was significantly smaller (0.067 vs. 0.081 m, *p* = 0.0012). Foot clearance wearing athletic shoes was significantly smaller on three of the four frequent paths (mean 0.030 vs. 0.035, *p* < 0.0001), with one exception on the sidewalk between the office building and dining location where the difference was not significant (*p* = 0.7797). The results in the hallway from the subject’s office to the restroom (path (ii) in Figure 6) are shown in Figure 7 as an example. 

**Figure 8:**
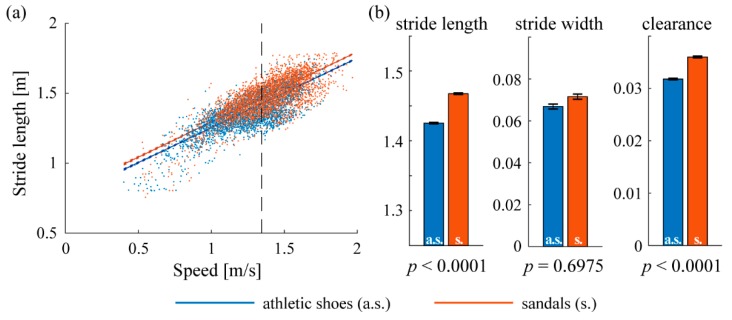
Spatiotemporal gait parameters using all straight-line walking bouts longer than 15 m. All conventions are similar to Figure 7. (**a**) Linear regression of stride length on walking speed showing the mean speed (1.34 m/s) where population marginal means of the two conditions are compared. (**b**) Population marginal means of stride length, stride width, and clearance; error bars indicate 95% confidence intervals.

We also performed a similar comparison of stride length, width and clearance across footwear conditions using all straight paths longer than 15 m (i.e., all paths shown in Figure 5). Figure 8 shows the ANCOVA results using this larger set; it can be compared to Figure 7 to observe the differences in outcome relative to the restricted set of frequently-repeated paths. Using all straight walking, stride length wearing athletic shoes was significantly shorter (mean 1.43 vs. 1.47 m, *p* < 0.0001), stride width was significantly smaller (mean 0.067 vs. 0.072, *p* < 0.0001), and foot clearance was significantly lower (mean 0.032 vs. 0.036, *p* < 0.0001) as well. Mean speed across all strides recorded on straight paths was 1.34 m/s. 

**Figure 9:**
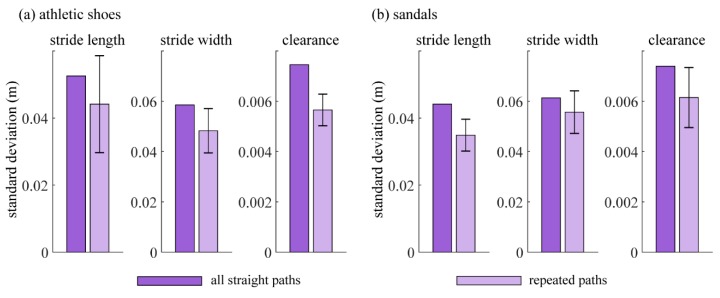
Comparison of variability of the spatiotemporal gait parameters between all straight paths and repeated paths when wearing (**a**) athletic shoes and (**b**) sandals. Dark columns indicate the variability of each outcome metric using all straight paths over 15 m (standard deviation across strides within 2% of the mean speed). Light columns with error bars indicate the variability of each outcome using only frequently-repeated paths (mean and standard deviation across frequent paths of the standard deviations of the individual paths using strides within 2% of the mean speed).

Finally, because the goal of the repeated paths was to reduce variability in the data, we compared the standard deviation of each outcome for strides within 2% of the mean speed, using repeated paths vs. using all straight paths longer than 15 m. Figure 9 shows that variability in the data is reduced using repeated paths for all outcome measures. No statistical comparison is possible because there is only one value for the all-straight-paths case. 

## 4. Discussion

### 4.1. Utility and Applicability of the Method

This preliminary result demonstrates the promise of the proposed method to identify frequently-repeated walking paths from wearable measurements across multiple days. The paths identified include both indoor hallways and outdoor sidewalks, potentially enabling the study of various daily settings that may have different contextual influences on subjects. Focusing on frequently-repeated paths may eliminate many confounds that cannot be excluded in typical activity-tracking studies, such as unique events that occur within the study window (e.g., running a race, attending a festival, touring a museum, and riding a cycle [3]); these unique activities are eliminated automatically by the repeated-paths selection process. The result is a set of focused activities that are more likely to be comparable across experimental conditions, enabling meaningful statistical comparisons of specific explanatory variables of primary interest such as different features of prostheses or surgical vs. rehabilitative interventions for injury. 

The relative benefits of using only frequently-repeated paths or all straight-line walking paths are revealed by Figure 7, Figure 8 and Figure 9. The presence of statistical differences in the comparison using all straight walking (Figure 8) illustrates the potential value for that simpler approach. Indeed, related all-strides analyses have been found useful in some prior work (e.g., Reference [12]). However, the repeated-paths analysis shows a benefit in reducing the variability in the data compared to using all straight paths (Figure 9). This reduced variability is likely attributable to greater similarity among the limited bouts included in the repeated-paths analysis. Reduced variability may enable improved sensitivity to differences across experimental conditions. The repeated-paths analysis also has the *a priori* conceptual benefit of eliminating unique behaviors or events from the analysis, thereby ensuring a defensible “apples-to-apples” comparison among conditions, much as in controlled laboratory studies. Comparison of Figure 5 and Figure 6 illustrates the unique or infrequent paths that were excluded. Among these are several that occurred only in one condition that could potentially bias the results of an all-strides analysis. Further application of the proposed repeated-paths analysis should continue to include comparison against all-strides analysis to further clarify their relative utility. 

The present study highlights straight-line walking, but further curve matching algorithms could be used to find repeated turning, stairs or ramps, which are seldom studied quantitatively but are important in assessing clinical interventions. In addition, the IMU itself is a rich resource for gait analysis, providing much finer spatiotemporal and kinematic gait parameters than a typical step counter [1,17,18,19,20,25,35]. The key finding is that the method enables detailed comparisons with many samples in semi-controlled data sets—an approach that could be powerfully applied to evaluate interventions such as prostheses, orthoses, surgeries, or medications. 

The subtle differences in walking with athletic shoes vs. sandals are not as dramatic nor as important as clinical interventions are likely to be. Nevertheless, these two types of footwear are commonly used and the preliminary finding of differences between them may motivate further study of how humans accommodate different footwear. We speculate that the two main findings—greater clearance and longer stride length with sandals—may be related to the fear of tripping or stubbing the toes. We suspect that clearance is increased deliberately to prevent the sandal from catching on obstacles, which is more likely to occur and to result in foot injury when wearing sandals. We further speculate that once the foot is lifted, the longer stride is simply a consequence of inertia: the foot travels forward at the same rate, but takes longer to reach the ground, resulting in a longer stride. More detailed outcome metrics such as toe and heel clearance [20] (rather than the IMU’s estimated stride clearance measured here) would be useful to better understand these effects. This single-subject study cannot definitively prove the effect nor its cause, but the potential mechanism is relevant to clinical applications such as foot drop and lower-limb prosthetics and orthotics. 

The proposed method can be extended to study outcomes beyond those captured by the foot-mounted inertial sensors. Any other sensor could be recorded synchronously and analyzed during comparable bouts of movement based on this location reconstruction. Potential examples include heart rate, electromyographic activity, plantar pressure distribution, prosthetic socket load, postural control of the trunk, gait symmetry, respiration rate, use of an impaired arm for manipulation, and more. Additionally, the proposed method could be used to infer the locations the subjects have visited during the test with a finer resolution than GPS, especially indoors. These location histories could be used in some social activity measurements to build up Location-Based Social Networks aimed at providing location-based services [36] or interventions against negative behaviors [37].

The method as presented was mainly designed to enable scientific comparison of different interventions, but it could also be extended to assess the performance or capacity of individual patients in a clinical context. For scalable deployment in the clinic, issues of cost, compactness, longevity and interpretability are of paramount concern. The system as used in this study is already sufficiently simple, adding only an inexpensive GPS and barometer compared to commodity IMU’s; it mainly needs miniaturization and automation of the processing. The frequent-paths analysis has potential clinical utility for improved comparison of individuals against normative data or for repeated-measures comparisons such as tracking recovery or decline over time. The method may also be extended to study movement across different types of terrain; for example, many persons with lower limb amputation walk well on smooth level ground, but their stability on uneven surfaces like grass or gravel varies widely. Wearable sensor data on spontaneous movement in all these environments could be used with the proposed clustering methods to identify paths that differ only in terrain. Then, the response of an individual to differences in terrain could be a tool for assessing his/her overall movement capacity. 

Finally, the location-aware data sets resulting from this method have the potential to provide unique information on unexpected events that occur in daily life, such as falls. For example, a recent study recorded motion from older adults for two weeks and reconstructed body movement surrounding the losses of balance they reported [23]. The proposed method uses a similar reconstruction but could extend the analysis to compare these events against movements in the same location that did not result in loss of balance. This approach could enable improved understanding of the circumstances that lead to falls.

### 4.2. Limitations and Future Work

As a dead reckoning navigation method, the trajectories reconstructed from IMU data do inevitably suffer from some limitations. For example, indoor parking lots can corrupt the subsequent indoor localization, because of the extended absence of available corrections (neither GPS nor ZUPT can be applied during driving and GPS may not be reestablished prior to entering a building). The same thing happens for walking or running on a treadmill in a gym, and other indoor activities during which the ZUPT assumption is violated. Identifying and handling these special cases remains a challenge for further algorithm development. Additionally, despite the success of the Kalman smoother technique, heading drift still poses a substantial threat to the accuracy of the trajectory, especially during long indoor periods with high activity level. The simple (but costly) way of handling it is to use an angular rate gyroscope with lower drift error. Ongoing research aims to further correct heading drift. Some potential approaches are based on adding hardware, such as placing Bluetooth beacons at the subject’s most frequented places (e.g., office desk) for absolute position correction. Other improvements are algorithmic, such as Foot SLAM (simultaneous localization and mapping) [38], which uses past trajectories as a map to constrain future trajectories, and other SLAM techniques to further improve the location estimate such as using ambient WiFi network signals [39] or magnetic field fluctuations [40]. Additional improvements may also refine the inertial reconstruction, such as tilt angle corrections based on gravity [19]. Fortunately, the proposed method generally rejects faulty reconstructions from statistical analysis because they are not frequently repeated. 

Another limitation of the proposed method is that there is no ground truth to verify the clustering of spatially repeated trajectories. Two types of errors may occur in the clustering: (i) Some indoor trajectories with large drift may not be grouped into the cluster where they are supposed to be and (ii) worse, some trajectories may be wrongly grouped into a cluster. Unfortunately, there is no way to discover these mistakes. However, proper thresholds and grouping methods can balance the two errors. For example, tightening the clustering criterion such that each candidate trajectory must have two or more other close trajectories in the same cluster (we used one in this study) would bias the errors toward the first type—a more conservative strategy for making meaningful comparisons. 

The structure of indoor movement in multi-level buildings provides additional challenges and opportunities for error management. Indoor trajectories are usually constrained by hallways, so heading drift is unlikely to cause faulty clustering since there is little chance for the drifted trajectory to be aligned with other hallways just right. On the other hand, hallways on different floors are usually aligned with each other, so vertical position drift can easily cause faulty clustering. Efforts to limit vertical position drift are ongoing, such as assuming that indoor walking does not change altitude and using additional measurements such as the barometric altitude data to detect ramps and stairs. 

The repeated-paths technique relies on the assumption that frequently-repeated movement paths imply repeatable circumstances during locomotion. This assumption is clearly imperfect, as common variations such as carrying a backpack or package, walking with other people, navigating crowds or encountering rain can occur on even the most-frequented paths. These variations are expected to be less common and less severe in restricted clusters of repeated paths than in larger data sets like “all movement”. However, the current set of sensors has no way to detect and reject these circumstances, so their presence could increase variability in the data. Future improvements may pursue a range of techniques to reject specific variations, such as using pressure insoles to detect load carriage, microphones to detect the wearer’s speech (implying accompaniment), LIDAR or step-frequency-based classification [41] to detect a crowd, or weather records to detect weather variations.

## 5. Conclusions

The proposed technique for analyzing movement along frequently-repeated paths harvested from long-duration wearable monitoring promises to enable studies of mobility patterns and interventions with laboratory-like precision, but without the artificial laboratory environment. The movements thus studied are by definition the most common movements an individual performs and are therefore arguably the most impactful movements to understand. By analyzing these common movements before vs. after an intervention or across multiple interventions, outcomes can be assessed to determine the comparative benefits of the interventions. This technique can potentially be extended to analyze specific targeted movements such as stairs or ramps; broadened to assess whole families of movements such as all indoor straight level walking; or adapted to compare movement features across different indoor and outdoor terrains.

## Figures and Tables

**Table 1:** Correspondence between different movement conditions and Kalman filter corrections.

Movement Conditions	Indoors			Outdoors	
	Stationary	Elevator	Others	Vehicle	Others
Kalman filter corrections	ZUPT, ZARU	Horizontal ZUPT, BA	ZUPT	GPS	GPS, ZUPT

## Supplementary Materials

The following are available online at https://www.mdpi.com/1424-8220/19/8/1925/s1.

## Appendix A. Recognition of Indoor vs. Outdoor GPS

To determine if a GPS fix is outdoors, we examine its preceding 20 fixes and subsequent 19 fixes and itself. If the following criteria are all met, the fix is recognized as outdoors: (i) at most 2 of the 40 fixes have HDOP larger than 10 (dimensionless); (ii) the average time gap between consecutive fixes is less than 3 s (GPS is sampled at 1 Hz); (iii) the maximum spatial separation between consecutive fixes is smaller than 30 m; (iv) mean speed is larger than 0 m/s; (v) if mean speed is smaller than 0.8 m/s, then either the nearest building is not the same for all 40 fixes or the mean distance from the nearest building is larger than 10 m and (vi) if mean speed is larger than 0.8 m/s, then either the nearest building is not the same for all 40 fixes or the mean distance from the nearest building is larger than 0 m. The mean speed is the average speed calculated from consecutive fixes. The distance between a fix and a building is defined as the shortest distance from the fix to the edges of the building if the fix is outside and 0 if the fix is inside of the building’s footprint.

## Appendix B. Kalman Filter and Smoother

### Appendix B.1. Structure of the Kalman Filter

In this appendix, the Kalman filter and smoother used in the proposed method are detailed. The structure of the Kalman filter is referenced from [42], but the differences are minor compared to [26,27]. The method involves two sets of states: nominal states and error states. Nominal states $x={\left[\begin{array}{ccccc} r^{T} & v^{T} & \theta^{T} & a_{b}^{T} & \omega_{b}^{T} \end{array}\right]}^{T}$ represent position, velocity, orientation, accelerometer bias and gyroscope bias integrated from IMU measured angular velocity and linear acceleration. Nominal states are corrected by the output of the Kalman filter when applicable corrections arrive. Error states $\delta x={\left[\begin{array}{ccccc} \delta r^{T} & \delta v^{T} & \delta \theta^{T} & \delta a_{b}^{T} & \delta \omega_{b}^{T} \end{array}\right]}^{T}$ are the estimated errors of the nominal states and are where the Kalman filter operates. Here the error of orientation is represented by rotation vector $\delta \theta =\|\delta \theta \|\cdot \vec{n}$, which is the rotation about axis $\vec{n}$ by an angle ‖δθ‖ in world-fixed coordinates. The linearized transition matrix for the error states is given by

(A1) $$F=\left[\begin{array}{ccccc} I_{3\times 3} & I_{3\times 3}\;\Delta t & 0 & 0 & 0\\ 0 & I_{3\times 3} & -{\left[R\left(a_{m}-a_{b}\right)\right]}_{\times }\;\Delta t & -R\;\Delta t & 0\\ 0 & 0 & I_{3\times 3} & 0 & -R\;\Delta t\\ 0 & 0 & 0 & I_{3\times 3} & 0\\ 0 & 0 & 0 & 0 & I_{3\times 3} \end{array}\right],$$

where $I_{3\times 3}$ is the 3-by-3 identity matrix, Δt is the sampling period, R is the rotation matrix from IMU coordinates to world-fixed coordinates, $a_{m}$ is measured linear acceleration, $a_{b}$ is estimated accelerometer bias, and ${\left[•\right]}_{\times }$ is the skew operator which produces the cross product matrix [42]. The covariance matrix P of the error states is iterated by the standard equation $P\leftarrow FPF^{T}+Q$, where Q is the covariance matrix of IMU’s noise estimated from product specifications or experiments.

Whenever applicable corrections arrive, such as GPS and ZUPT, a measurement matrix H selects the corresponding states. In the case of GPS and ZUPT, H is given by

(A2) $$H=\left[\begin{array}{ccccc} I_{3\times 3} & 0 & 0 & 0 & 0\\ 0 & I_{3\times 3} & 0 & 0 & 0 \end{array}\right].$$

The Kalman gain is calculated by $K=PH^{T}{\left(HPH^{T}+V\right)}^{-1}$, where V is the covariance matrix of the measurement noise which can be optimized for best accuracy. The error states are updated by δx=K(y−Hx), where y is the measurement, in the case of GPS and ZUPT, $y={\left[\begin{array}{cccccc} r_{x,\mathrm{GPS}} & r_{y,\mathrm{GPS}} & r_{z,\mathrm{GPS}} & 0 & 0 & 0 \end{array}\right]}^{T}$. The posterior covariance matrix is then updated by $P\leftarrow \left(I_{15\times 15}-KH\right)P$. Finally, the nominal states are corrected by the error states, x←x+δx. Note that though “+” is used here, the correction of orientation involves applying the error rotation to the nominal orientation rather than a simple addition [42].

### Appendix B.2. Kalman Smoother for Location: Forward and Backward Kalman Filters

The Kalman smoother makes use of the fact that the covariance matrix P is an estimate of how accurate the nominal states are. The same Kalman filter and integration process are run backward from the last sample to the first sample. The only difference is the sampling period Δt is replaced by its negative, −Δt. The forward and backward Kalman filters produce two posterior covariance matrices at a timestamp, $P^{+}$ and $P^{-}$, which are used to average the nominal states $r^{+}$ and $r^{-}$ [28] component-wise by

(A3) $$r_{x,\mathrm{smoothed}}=\frac{r_{x}^{+}/\mathrm{var}(r_{x}^{+})+r_{x}^{-}/\mathrm{var}(r_{x}^{-})}{1/\mathrm{var}(r_{x}^{+})+1/\mathrm{var}(r_{x}^{-})},$$

where $\mathrm{var}(r_{x}^{+})$ and $\mathrm{var}(r_{x}^{-})$ are the left-top entry of $P^{+}$ and $P^{-}$ respectively, which are the estimated variances of the position in *x*-axis. Position in *y*-axis and *z*-axis are averaged in the same fashion. The intuitive description of this process is that forward and backward positions are averaged according to their estimated accuracy to produce the smoothed position.
