# Supplementary figures and images for: Analyzing Gait in the Real World Using Wearable Movement Sensors and Frequently Repeated Movement Paths
Source: Sensors (Basel). 2019 Apr 24;19(8):1925. doi: 10.3390/s19081925 (PMC6515355; doi:10.3390/s19081925)

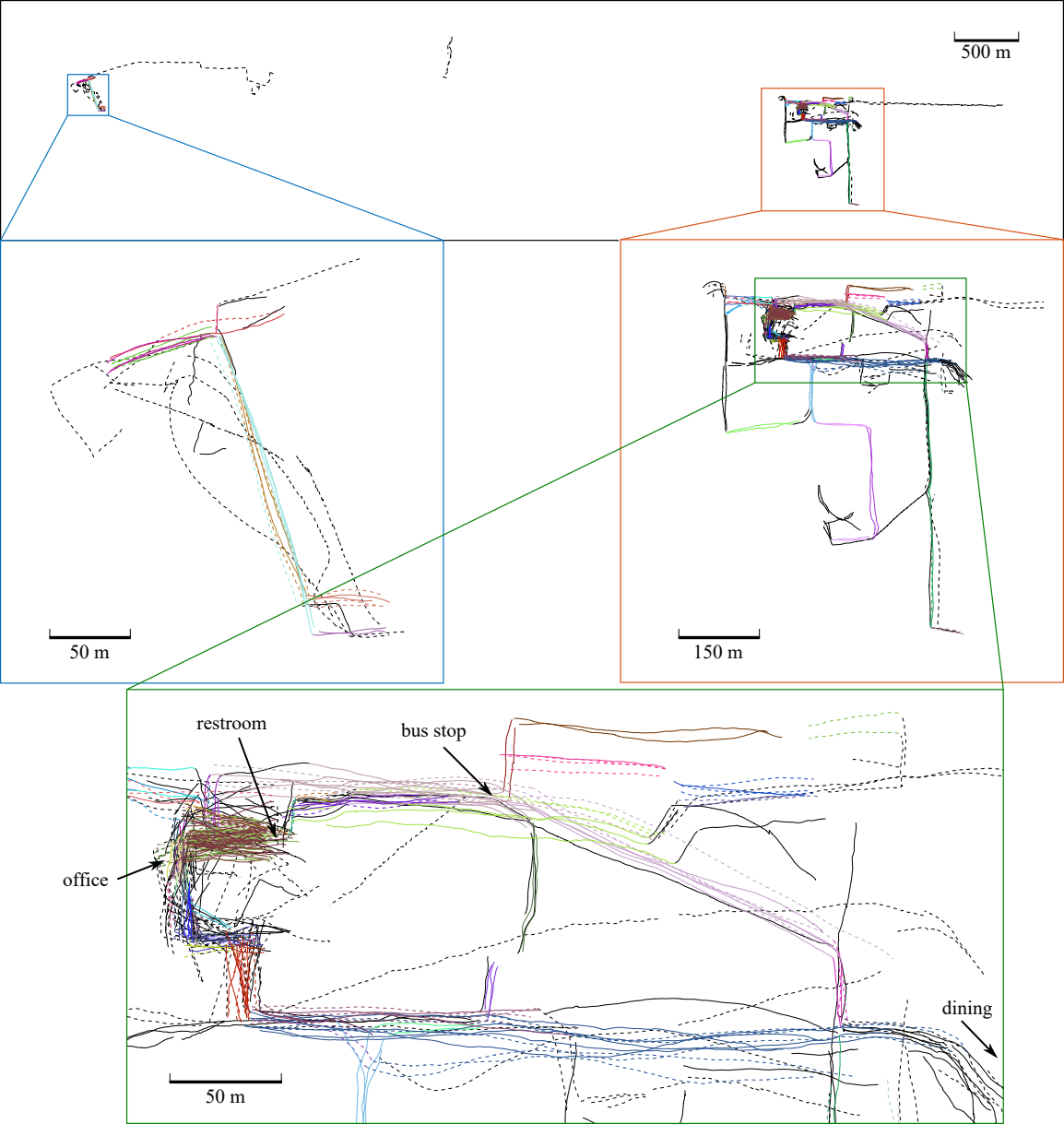

Supplement: Supplementary file 1 [file sensors-19-01925-s001.zip › Supplement - Fig 5 in SVG and PDF/Supplement_FIG5_PDF.pdf]
